# Evaluation of canine prostate volume in calculated tomographic images - comparison of two assessment methods

**DOI:** 10.1186/s12917-019-2106-3

**Published:** 2019-10-22

**Authors:** Katharina Haverkamp, Lisa Katharina Harder, Nora Sophie Marita Kuhnt, Matthias Lüpke, Ingo Nolte, Patrick Wefstaedt

**Affiliations:** 1 Small Animal Clinic, University of Veterinary Medicine Hannover, Foundation, Bünteweg 9, D-30559 Hannover, Germany; 2 Institute for General Radiology and Medical Physics, University of Veterinary Medicine Hannover, Foundation, Bischofsholer Damm 15, House 102, D-30173 Hannover, Germany

**Keywords:** Prostate, Computed tomography, Dogs, Volume, Amira, Slice addition technique, Formula, Wrap

## Abstract

**Background:**

Since most prostatic diseases are associated with the organ’s enlargement, evaluation of prostatic size is a main criterion in the diagnosis of prostatic state of health. While enlargement is a non-uniform process, volumetric measurements are believed to be advantageous to any single dimensional parameter for the diagnosis of prostatomegaly. In a previous study, volume was analysed with a slice addition technique (SAT), which was validated as highly accurate. Irrespective of high accuracy, SAT represents a complex and time-consuming procedure, which limits its clinical use. Thus, demand exists for more practical volume assessment methods. In this study, the prostatic volume of 95 canine patients (58 intact males, 37 neutered males) were analysed retrospectively by using the ellipsoid formula (Formula) and an imaging “wrap” function tool (Wrap) to help assess accuracy and applicability. Accuracy was checked against phantom measurements and results were compared to SAT measurements of the same patient pool obtained from a previously published paper. Patients were grouped according to prostatic structure (H = homogeneous, I = inhomogeneous, C = cystic) and volume using the SAT (volume group = vg: 1, 2 and 3).

**Results:**

High correlation between the Formula or Wrap volume and the phantom volume was found, the values being higher for the Formula. Mean Formula volumes (vg 1: 2.2 cm^3^, vg 2: 14.5 cm^3^, vg 3: 109.4 cm^3^, respectively) were significantly underestimated, while mean Wrap volumes (vg 1: 3.8 cm^3^, vg 2: 19.5 cm^3^, vg 3: 159.2 cm^3^) were statistically equivalent to SAT measurements (vg 1: 3.1 cm^3^, vg 2: 18.6 cm^3^, vg 3: 157.2 cm^3^, respectively). Differences between Formula and SAT volumes ranged from 22.4–31.1%, while differences between Wrap and SAT volumes were highest in small prostates (vg 1: 22.1%) and fell with increasing prostatic size (vg 3: 1.3%).

**Conclusion:**

The Wrap function is highly accurate, less time-consuming and complex compared to SAT and could serve as beneficial tool for measuring prostatic volume in clinical routine after further validation in future studies. The Formula method cannot be recommended as an alternative for volumetric measurements of the prostate gland due to its underestimation of volumes compared to SAT results.

## Background

Diseases of the prostate gland are common, especially in older intact male dogs, and in most cases enlargement of the organ is seen [1]. Indeed, symptoms are non-specific like prostate fluid discharge, abdominal pain, or stiffness in the hind limbs, which makes diagnosis of prostatic diseases difficult [2]. In human medicine, the prostate-specific antigen (PSA) was found to be a useful prostatic biomarker for early stage diagnosis of prostatic diseases. In dogs, the canine prostate specific esterase (CPSE) was observed to follow similar hormonal metabolisms like PSA in men. In blood samples, measuring CPSE levels can be used to determine prostatic diseases, where prostate size is 1.5 times the size of the estimated normal size of the prostate gland. CPSE levels over 50 ng/mL are associated with prostatic diseases [3]. Indeed, no differentiation between benign prostatic hyperplasia, prostatitis, prostatic cysts and prostatic carcinomas is possible. Since in most prostatic diseases enlargement of the gland is visible, investigating prostatic size has become the main criterion in evaluating prostatic state of health [1]. Due to the fact that prostatic enlargement has been observed to be non-uniform, being greater in length than height [4], one-dimensional parameter measurements might lead to misinterpretation of prostatic size. Thus, volumetric measurements, combining single dimensional parameters might be advantageous for diagnosing prostatic enlargement. Determining prostatic volume was reported to be an accurate method for describing alterations in prostatic size and helpful for differentiating between different prostatic alterations [5]. The volume of cystic prostates was significantly higher compared to that of homogeneously structured prostates in computed tomography (CT) images. Thus, volumetric measurements seem to be beneficial for the diagnosis of prostatic health status. Therefore, they might represent a valuable diagnostic procedure in clinical routine in addition to digital rectal palpation, x-ray and ultrasound examination of the prostate. To date, most studies have investigated prostatic volume formulas with measurements of length, height and width in ultrasonography, resulting in over- or underestimation of real prostatic real volume [6–10]. Nevertheless, ultrasound represents the method of choice for investigating prostatic diseases in dogs. It is an easy to perform and highly specific diagnostic tool for investigating the prostate gland. With ultrasound guidance, fine needle aspiration biopsies are performed safely, resulting in accurate diagnosis [11]. Due to the absence of organ superimposition and less dependence on operator’s experience in comparison to ultrasonography, many studies recommended computed tomography (CT) imaging for investigating the size and tissue morphology of the prostate as well as for allowing characterisation of surrounding structures (e.g. sublumbar lymph node assessment) [7, 12–14], especially for scientific studies [11]. However, there is only scant literature available on volumetric measurements of the prostate gland in CT, which might be explained by the necessity of anaesthesia during examination and the limited access to computed tomography in veterinary medicine. Schulze et al. examined prostatic volume in CT datasets with a formula of an ellipsoid body without knowing the real volume of the examined prostates [15]. In former ultrasound studies the formula-based determination of prostatic volumes was found to be inconsistent due to over- or underestimation of volume [6, 8, 10]. Whether the same over- or underestimation occurs when CT datasets are used has yet to be investigated. Choi et al. and Lee et al. examined prostatic volume by means of CT data with a rendering software tool, but accuracy was not validated [7, 13]. The slice addition technique (SAT) is recommended as a beneficial and highly accurate tool for measuring organ volumes in dogs [16] and humans [17]. Moss et al. who measured volume of the canine liver, kidney and spleen with this procedure showed that SAT was accurate to +/− 5% of the organ volume measured by water displacement [16]. Nevertheless, measurements of the prostate gland were not included in their study. A previously carried out study by Haverkamp et al. applied this technique to measure the volume of the canine prostate gland for the first time [5]. Comparison of SAT measurements to real volumes of different phantoms and cadaver prostates exhibited a high accuracy with a variation of ±0.8% to real volume. However, SAT requires the marking of all transversal slices of the prostate gland. This is a complex and time-consuming procedure which rules out implementing this technique for routine clinical practice. The usage of an ellipsoid formula is reported to be less complex for measuring the prostatic volume in ultrasound [9], but was not validated in CT. Furthermore, we assumed that automatic volume assessment methods are less complex procedures. Thus, the aim of this study was to evaluate 1) an ellipsoid formula-based assessment method and 2) a nearly automatic function tool called Wrap to determine canine prostatic volume in CT with regard to clinical feasibility and accuracy. On the one hand, the accuracy of these volumetric measurements was validated with phantoms and cadaver prostates with known volume. On the other hand, measurements were compared to SAT measurements of the same images carried out in a previous study [5]. Since the different size and surface structure of the prostates may have an impact on the accuracy of measurement methods, CT data should be evaluated taking different categories of prostatic sizes as well as the castration status of the dogs and the prostate tissue structure into consideration.

## Results

**Fig. 1:**
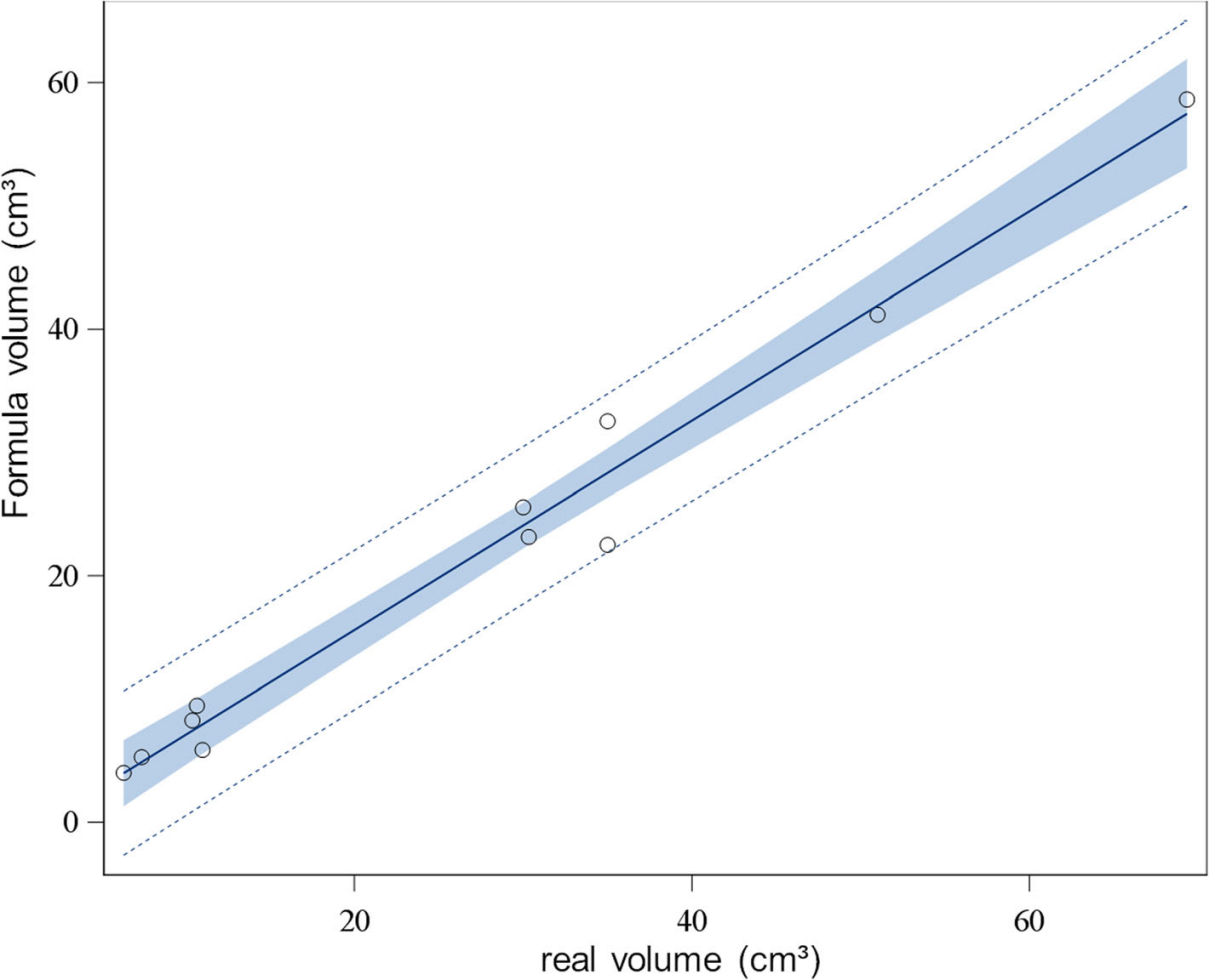
Linear regression analysis of Formula-derived and real-volume phantom measurements. Dotted lines represent 95% prediction limits, blue area represents 95% confidence limits

**Fig. 2:**
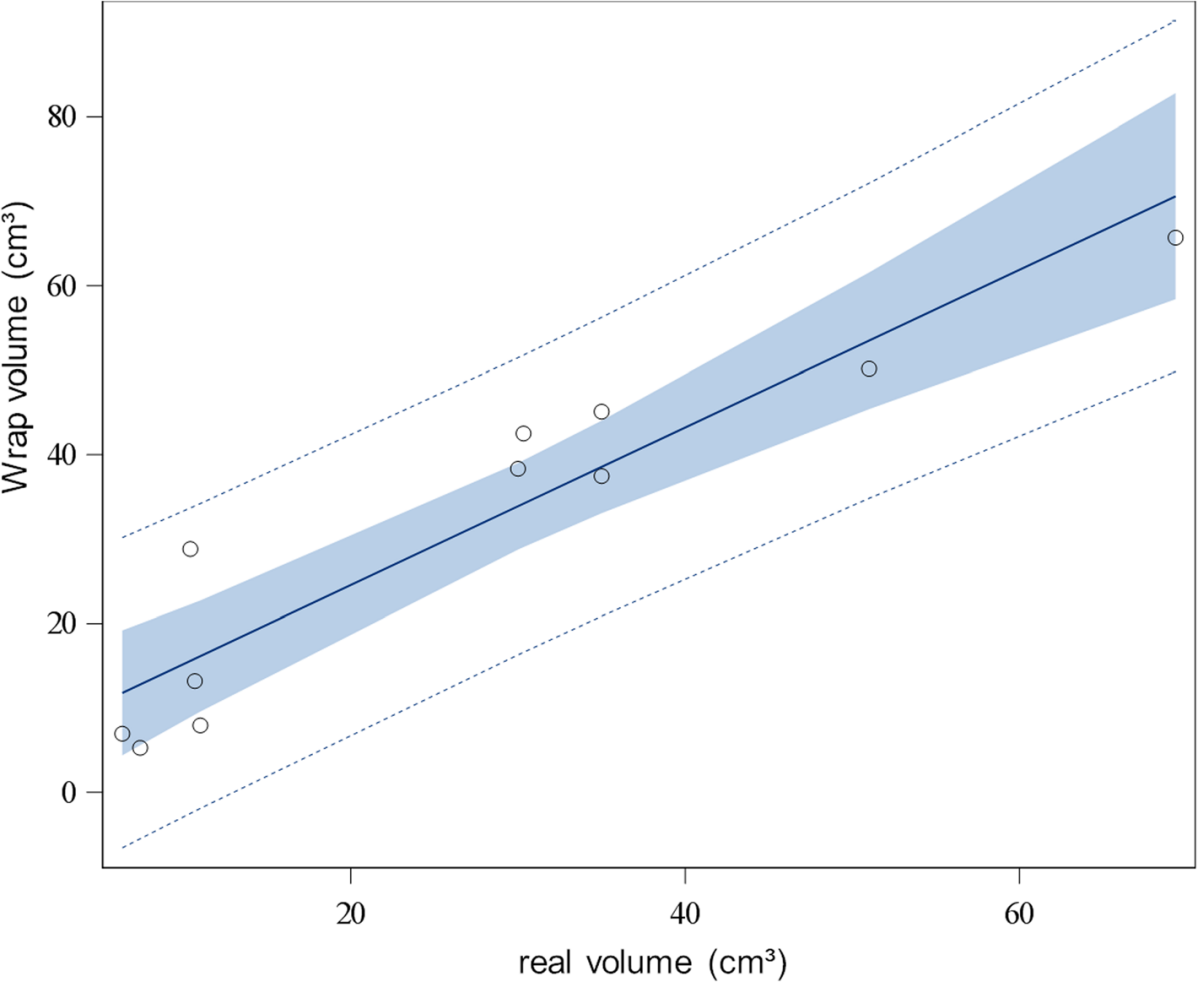
Linear regression analysis of Wrap-derived and real-volume phantom measurements. Dotted lines represent 95% prediction limits, blue area represents 95% confidence limits

Linear regression analysis exhibited a significant correlation between real volumes of the phantom measurements and Formula- and Wrap-derived volumes (Formula: *p* < 0.001, Wrap: p < 0.001, respectively). The R-square value was 0.98 for the Formula method and 0.88 for the Wrap function (Figs. 1 and 2).

**Fig. 3:**
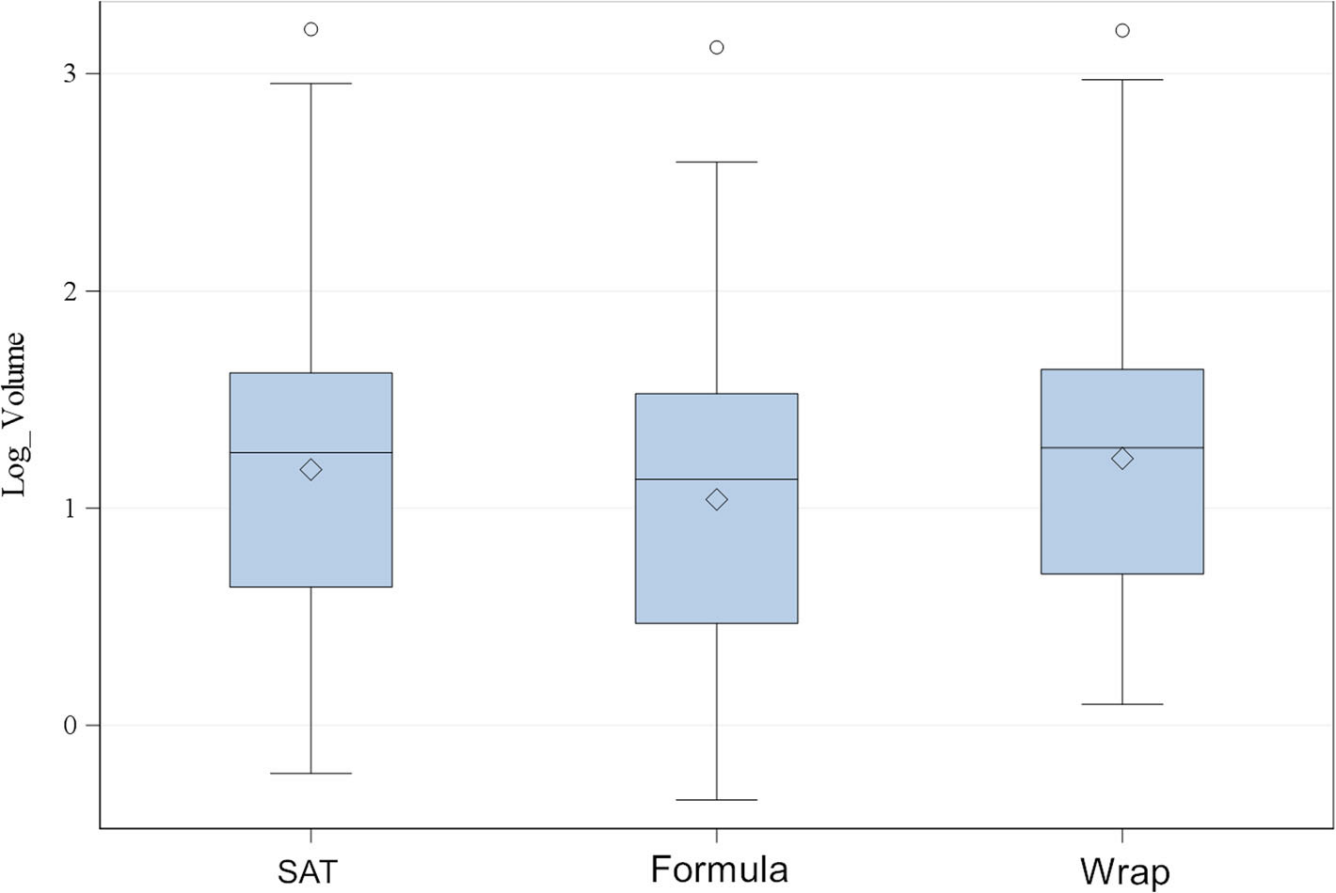
One-way analysis of prostate volume of all analysed dogs using different assessment methods. For statistical analysis, the volume was logarithmised. Values of the slice addition technique (SAT) were obtained from a previously published study [3]

The mean volume measured by the Formula method was 41.3 cm^3^ (± 141.4 cm^3^, ranging from 0.5 to 1319.8 cm^3^) and the mean volume measured by the Wrap function was 59.8 cm^3^ (± 188.7 cm^3^, ranging from 1.3 to 1579.2 cm^3^). The SAT prostate volume values had been obtained from a previous study investigating the same dogs [5]. The mean SAT prostate volume was 58.6 cm^3^ (± 188.6 cm^3^, ranging from 0.6 to 1600.5 cm^3^). There were no statistically significant differences between the three methods (Fig. 3), although the mean volumes differed: the Formula volume was 29.5% lower compared to the SAT and the Wrap was 2.1% higher compared to the SAT. The mean volume values separated into volume groups 1, 2 and 3 are shown in Table 1.

**Table 1:** Mean volume values (± SD) of SAT, Formula method and Wrap function separated into volume groups

Assessment method/function	Mean volume (± SD) of vg 1	Statistical comparison	Mean volume (± SD) of vg 2	Statistical comparison	Mean volume (± SD) of vg 3	Statistical comparison
SAT (cm^3^)	3.1 (± 1.6)	A	18.6 (± 8.3)	A	157.2 (± 310.4)	A
Formula (cm^3^)	2.2 (± 1.2)	B	14.5 (± 7.0)	B	109.3 (± 235.4)	B
Wrap (cm^3^)	3.8 (± 1.7)	A	19.5 (± 7.8)	A	159.2 (± 310.2)	A

Significant differences are indicated by letters. Groups with the same letter are not significantly different. SAT volumes are derived from a previously published paper [3]

*SAT* slice addition technique, *SD* standard deviation, *vg* volume group

There were significant differences between SAT volumetric measurements and volumes measured by Formula in volume groups 1 (*p* = 0.026), 2 (*p* = 0.030) and 3 (*p* = 0.020). Differences were not significant between SAT- and Wrap-derived volumes in all three groups. Differences between Formula- and Wrap-derived values were significant in volume groups 1 (*p* < 0.001), 2 (*p* = 0.007) and 3 (*p* = 0.016).

The bias was determined as the mean difference of the SAT measured volume subtracted from the Formula- or Wrap-measured volume. Detailed results for the different assessment methods are shown in Table 2.

**Table 2:** Bias values (standard deviation) for Formula and Wrap-derived measurements depending on different volume groups

	Volume group 1	Volume group 2	Volume group 3
Formula (cm^3^)	0.9 (± 0.6)	4.1 (± 2.7)	47.9 (± 99.6)
Wrap (cm^3^)	- 0.7 (± 0.4)	- 0.9 (± 1.1)	- 2,0 (± 12.8)

**Fig. 4:**
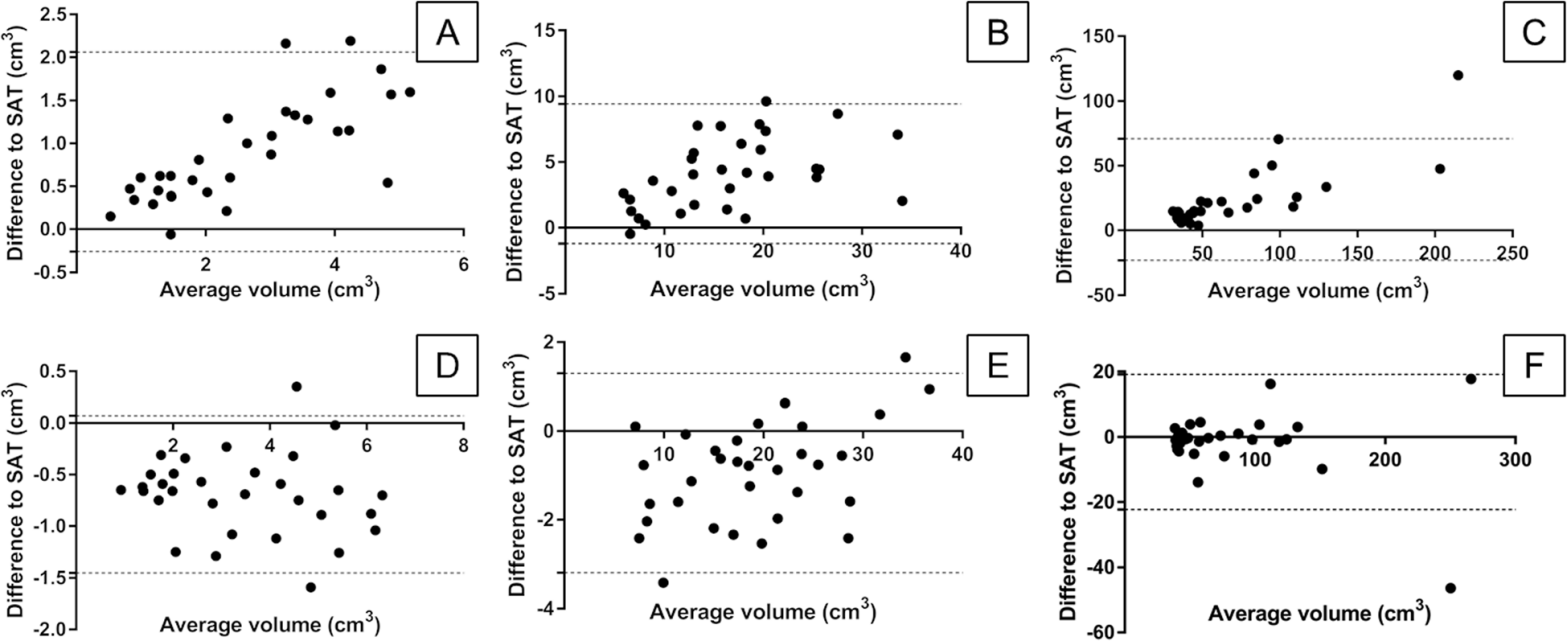
Bland-Altman Plot. **a-c** represent differences between Formula method to SAT in volume groups 1 (**a**), 2 (**b**) and 3 (**c**). **d-f** show differences between Wrap function and SAT in volume groups 1 (**d**), 2 (**e**) and 3 (**f**). For better visual analysis, the two highest values (outliers) were excluded from chart **c** and **f**. Due to different volume groups and varying discrepancies between the assessment method and function, the scaling of x- and y-axis differed between images **a-f**. SAT = slice addition technique

The Bias and the Bland-Altman plots showed a tendency of greater differences in Formula measurements by increasing prostatic volume within the volume groups (Fig. 4a – c), while the tendency in Wrap measurements was not as distinct (Fig. 4d – f).

**Fig. 5:**
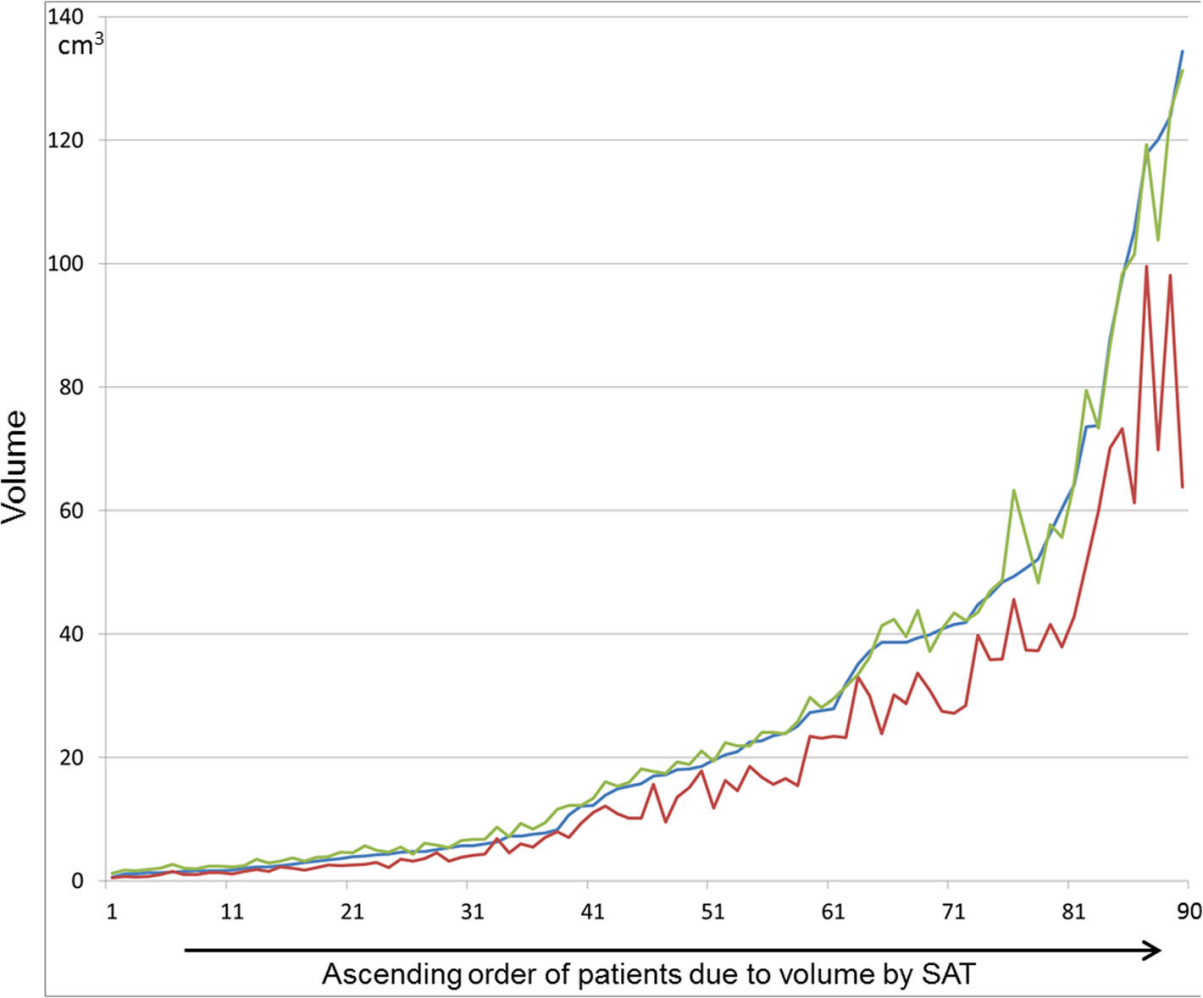
Comparison of volumes measured in SAT, Formula and Wrap. For better graphical presentation, the five highest values (outliers) were excluded. Dogs are plotted in ascending order on the x-axis according to their prostatic volume measured by SAT (y axis) (e.g. dog no.1: low volume, dog no. 90 high volume). Differences between SAT and Wrap-measured volumes are smaller than differences between SAT- and Formula -measured volumes. With increasing prostate volume, differences between SAT and Formula-derived volumes increase. Blue line: volume by SAT, green line: volume by Wrap, red line: volume by formula. SAT = slice addition technique

Tendency of greater differences in Formula measurements by increasing prostatic volume is also visible in Fig. 5. For better visual analysis, the five highest volume values have been excluded.

Percentage deviations between Formula and SAT were - 29.3, − 22.1% and - 30.4% for volume groups 1, 2 and 3, respectively. Percentage deviations between Wrap and SAT were 22.5, 5.1 and 1.3% for volume groups 1, 2 and 3, respectively.

## Discussion

In the past, many studies were carried out to determine prostatic size in dogs [6–10, 13–15, 18–21]. Usually, the size of the gland is determined by measuring single dimensional parameters like length, height and width, but these might lead to misinterpretation due to non-uniform enlargement of the prostate. Moss et al. hypothesised that evaluation of volume provides a more accurate measurement of the organ’s size (canine liver, kidney, spleen) than measuring the organ’s length and width [16]. In a previously published paper, volumetric measurements provided more information on prostatomegaly than single dimensional parameters were able to [5]. Furthermore, it could be shown that analysing prostate volume exhibited the potential to demonstrate prostatic alterations caused by castration, age, body size and different prostatic structures. By volumetric measurements, it was possible to differentiate between normal prostrates and those with alterations (e.g. cystic or inhomogenous tissue structure) in castrated dogs as well as between already altered prostates in intact male dogs. Thus, evaluation of prostatic volume was recommended as a beneficial tool in the diagnosis of prostatic diseases. To date, most studies have focused on the use of ultrasound to determine prostatic volume [6, 8, 18–21]. Contrary to ultrasound, CT is reported to be less dependent on the operator’s experience [14]. However, the literature relating to the volumetric measurement of the canine prostate gland using computed tomography with application of a formula is sparse [15]. Schulze et al. measured prostatic volume in CT datasets by means of an ellipsoid formula, but real volume was unknown [15]. Other studies used a volume rendering software tool to analyse prostatic volume in CT, though accuracy was not checked [7, 13]. In a recently published paper, the SAT was found to be a highly accurate method for determining the prostate gland volume [5]. Disadvantages existed due to complexity and high expenditure of time [9, 10]. Thus, there is demand for a more practical procedure for determining prostatic volume [5]. Therefore, the aim of this study was to validate the Formula method and Wrap function using Amira software as two methods for calculating prostatic gland volume using CT with regard to accuracy and practical application. For this purpose, accuracy was evaluated by comparing these results with measurements of phantoms and cadaver prostates with known volumes. Furthermore, prostatic volume results from Formula and Wrap measurements were compared to SAT measurements from a recent study [5] as SAT is a highly accurate technique for determining the prostate’s real volume.

### Measurements based on the formula method

In the present study, the Formula method was well correlated with phantom measurements. The R-Square values of the Formula method and that of the SAT of a previous study were equal (R = 0.98). Despite good correlation in our study, the Formula method showed underestimation of phantom measurements and significantly underestimated the prostatic volume compared to SAT for all volume groups. This is in agreement with results of a study by Choi et al. who assessed prostatic volume from CT using a volume rendering software tool and from ultrasound images using two different formulas [7]. One of these formulas significantly underestimated prostatic volume compared to CT-derived volume, but the real volume was not known. Choi et al. explained these lower values by using different examination devices (CT and ultrasound) and by means of the ellipsoid formula [7]. The influence of different examination devices on volumetric measurement results was not analysed in the present study, investigations being exclusively performed on computer tomographic images.

Kälkner et al. compared volumetric measurements of men’s prostate by SAT in CT with measurements of an ellipsoid formula in transrectal ultrasound (TRUS) [9]. CT-derived values were 48% higher compared to the TRUS ellipsoid method. In the present study, volume measured by the SAT in CT was 41.9% higher compared to the formula-derived volume. Indeed, both methods were performed on CT images, while Kälkner et al. compared volumetric measurements in CT with measurements in ultrasound. Furthermore, observations in phantom and cadaver measurements showed that Formula-derived values were underestimated compared to real volumes. Differences between CT- and TRUS-derived volumes were explained by the patients’ positioning during examination [9]. Patients were examined in lithotomy position in ultrasound and in dorsal position in computed tomography. In the present study, patients were examined in either dorsal or ventral recumbency and the same scan dataset was used to measure prostatic volume with the three different assessment methods. Thus, in this study, differences between the volumetric measurement methods could not be explained by differences in the patient’s positioning.

In contrast to the present study, other authors reported that Formula-derived volume values were overestimated [8, 10]. Kamolpatana et al. compared prostatic volume measured by an ellipsoid formula in transabdominal ultrasound [8] with real volume determined by water displacement. Formula-derived values showed overestimation, but differences to real volume were insignificant. Even in the present study, volume values by Formula correlated well with real volume of phantom and cadaver measurements, while measurements of the prostate gland by means of the Formula method were significantly underestimated compared to SAT volumes.

In human male patients, Terris et al. compared prostatic volume measured by a formula of an ellipsoid body and several deviations of this method in transrectal ultrasound to prostatic weight obtained from radical prostatectomy or cystoprostatectomy [10]. Volumes determined by ellipsoid formula were overestimated in 90% of the cases. While application of the ellipsoid formula represented an easy alternative, errors occurred due to difficulties in determining cephalocaudal dimension, these being caused by ambiguous delineation in the junction between the urinary bladder and prostate gland and urethra [10]. Furthermore, Terris et al. did not compare calculated prostatic volume to real volume but to prostatic weight [10]. These two factors might also explain why results of the ellipsoid formula were overestimated, while measurements of the ellipsoid formula in the present study were underestimated. Another reason might be the different anatomy of the prostate gland between dogs and men [8].

### Measurements based on wrap function in computed tomography

In this study, a novel function called Wrap was analysed for the first time to measure the volume of the canine prostate gland. The Wrap function showed good correlation between calculated and real volume values by phantom and cadaver measurements, but the R-square value (0.88) was lower compared to the SAT (0.98) and Formula (0.98). Indeed, comparison to measurements of the prostate gland using the SAT exhibited a higher agreement than comparison of the SAT and Formula method. Measurements using the Wrap function were 2.1% higher compared to the SAT, while those using the Formula method were 29.5% lower compared to the SAT. Since volume of phantoms and cadaver prostates were low and percentage differences in the volume calculated using the Wrap function and SAT were highest in small-sized prostates this might explain why agreement in phantom and cadaver measurements was lower for the Wrap than for the Formula method.

### Impact of differently sized prostates

Differences between Formula-derived volume and volume calculated by the SAT tended to become higher with increasing size of the prostate gland in the present study. This is in agreement with the study by Terris et al. who found that measurements of smaller prostates (≤ 80 g) in humans were best realised by using a variation in a prolate spheroid formula {π/6 x (transverse diameter)^2^ x (anteroposterior diameter)}, while larger prostates (> 80 g) were best analysed using a formula for a sphere {π/6 x (transverse diameter)^3^} [10]. Higher differences within larger glands might be caused by alteration in prostatic shape with increasing size. In this study, similar results were found with high numbers of inhomogeneous and cystic prostates in volume group 3. The loss of the nearly ellipsoid shape seems to be the reason why an ellipsoid formula is not sufficiently adequate to measure volume in the case of larger prostates.

Due to larger total volumes, differences between SAT and Formula are not proportionally greater with increasing prostatic size. In volume groups 1, 2 and 3, Formula-derived volumes were - 29.3, − 22.1% and - 30.4% smaller than the SAT-derived ones. Thus, the Formula method was inappropriate to measure prostatic volume irrespective of prostatic size.

With the Wrap function, highest differences to SAT-derived volume were observed in small sized prostates. Hence, the Wrap function is able to present the actual prostatic volume irrespective of prostatic size, but shows weaknesses in analysing small-sized prostates, mostly observed in neutered dogs. Excellent agreement was achieved in large prostates with volume values ranging from about 39 to 1600 cm^3^.

### Limitations of formula method and wrap function

The Formula method is unable to account for paraprostatic cysts as long as these cysts are excluded from measurements of height, length or width. However, including paraprostatic cysts in the measurements would yield too high volume values as the volume results from the local extension of the paraprostatic cyst over the boundaries of the prostate gland.

The wrap function has similar disadvantages. If the manually drawn crosshair does not include the paraprostatic cyst, the organ volume might be underestimated. Including the cyst in the prostate leads to more accurate measurements.

### Possible application in clinical routine

Volumetric measurements using SAT were associated with high complexity and time consumption. Based on our experience, depending on prostate size, this procedure took between 15 and 150 min and is therefore unfeasible in clinical routine. In contrast, the Formula method and Wrap function were easy to perform. Since Formula measurements were repeated twice, expenditure of time was slightly higher (nearly five minutes) compared to the Wrap function (nearly two minutes). Although the Formula method is straightforward, deviations to the SAT measurements are too high (between 22.1 and 30.4%) to be able to determine the real volume of the prostate gland. As Wrap is highly accurate, easy to perform and fast it represents an excellent alternative to SAT and could thus be beneficial in clinical routine.

### Limitation

Since accuracy of the Formula method and Wrap function were validated with measurements of small-sized phantoms only, it remains to be investigated whether the same results would be obtained with phantoms of larger size. Thus, further studies should verify the accuracy of both Formula and Wrap against different sized phantoms and a larger number of cadaver prostates with different sizes. Formula- and Wrap-derived measurements of prostatic volumes were compared to volumes achieved by SAT from a previously published study [5] and not to the real volume of the respective prostates. Thus, small variations in comparison to the real volume of the prostates are possible. Since only one author measured the volume, further studies are needed to verify interobserver variability for future clinical application. Furthermore, the reading radiologist was blinded to the volume groups but was aware of prostatic structure. This could be a source of bias.

## Conclusion

Volumetric measurements can be a helpful instrument to evaluate prostatic state of health. In general, before being applied to clinical routine, volumetric measurements need to be evaluated further in future studies, including a large number of subjects with different age and prostatic disease. The Wrap function could be established as a promising alternative to the highly precise, but complex SAT for measuring the volume of the canine prostate gland. The Wrap function is highly accurate, less time-consuming and less complex compared to SAT and is therefore a beneficial tool for measuring prostatic volume in clinical routine. Indeed, the Formula method cannot be recommended as a suitable alternative for performing volumetric measurements of the prostate gland due to its distinct underestimation compared to the SAT volumes.

## Methods

### Patients

In this retrospective study, CT datasets of dogs presented to the Small Animal Clinic, University of Veterinary Medicine Hannover, Foundation, Germany from October 2007 to August 2017 were included. All patient owners signed an informed consent form to data protection that states that collected data can be used for scientific research. The inclusion criteria were as follows: male, aged eights month or older, abdominal CT examination with contrast agent, no imaging artifacts like high-density streaks from metal implants. The CT study had to include the whole prostate. Dogs that had been chemically castrated using hormone substitution were excluded from this study.

**Fig. 6:**
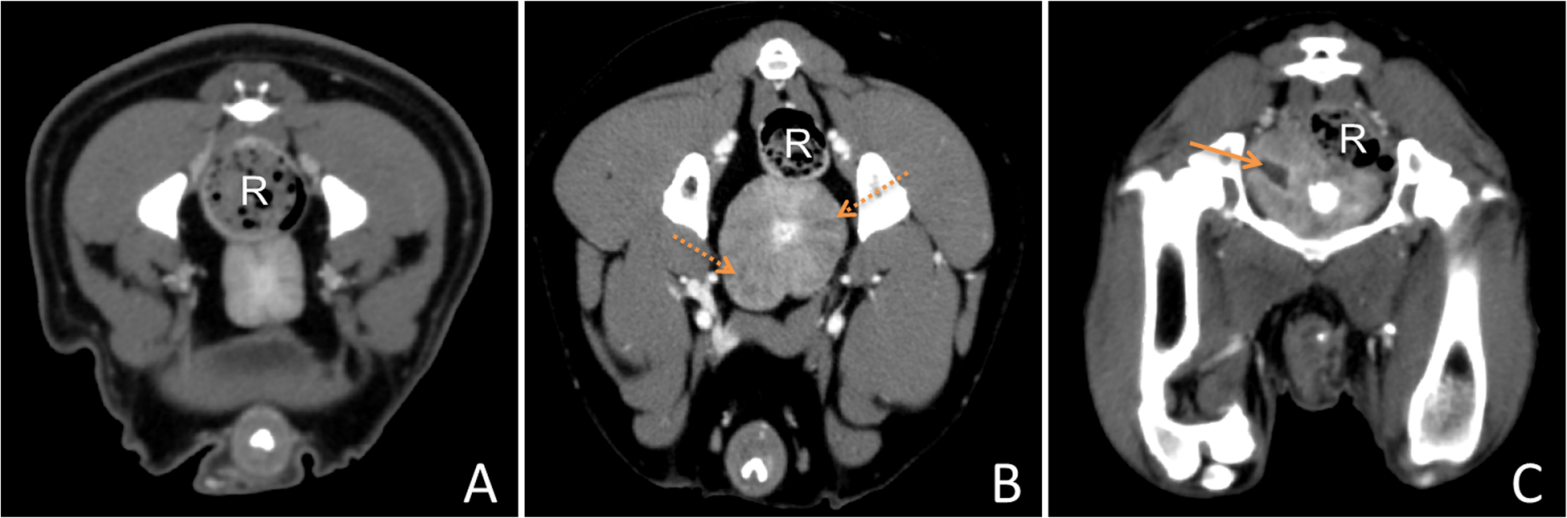
Different prostatic structures in CT images: **a** Homogeneous prostate, **b** Inhomogeneous prostate, **c** Cystic prostate, Dotted arrows indicate inhomogeneous parts of the prostate. Normal arrows indicate cystic alteration of the prostate. R = rectum

Ninety-five patients met the criteria. The mean age was 7.6 years and mean body weight 28.4 kg (Additional file 1). The study included 58 intact male (mean age 7.3 years and mean weight 30.8 kg) and 37 neutered male dogs (mean age 8.2 years and mean weight 25.5 kg). Patients were numbered serially according to increasing volume measured by SAT [5]. To sustain groups of equal size, patients 1 to 32 were assigned to volume group 1 (0–5.97 cm^3^), patients 33 to 64 were assigned to volume group 2 (6.32–37.15 cm^3^) and group 3 consisted of 31 patients with the highest volume values measured by SAT (38.58–1600.53 cm^3^). Detailed information on allocation of the dogs to the different groups is shown in Table 3. Furthermore, patients were grouped according to their castration status (intact, neutered) and according to the structure of the prostate tissue in CT images as follows: homogenous tissue (H), inhomogeneous tissue (I) and cystic tissue (C; diameter of cysts ≥1.2 mm) (Fig. 6). Each prostate was analysed by the same observer (first author).

**Table 3:** Patients’ allocation to different groups

Volume group	Range of volume by SAT (cm^3^)	Number of patients	Group structure		Status of castration (i/n)
1	0.60–5.97	32	H	23	3 / 20
			I	6	2 / 4
			C	3	0 / 3
2	6.32–37.15	32	H	2	2 / 0
			I	8	2 / 6
			C	22	18 / 4
3	38.58–1600.53	31	H	1	1 / 0
			I	5	5 / 0
			C	25	25 / 0

*SAT* slice addition technique, *H* homogeneous, *I* inhomogeneous, *C* cystic, *i* intact male, *n* neutered male

### CT data acquisition

A 64-multi-detector-row CT scanner (Phillips Brilliance 64, Philips GmbH, Hamburg, Germany) was used for abdominal CT scans at the Small Animal Clinic of the University of Veterinary Medicine Hannover, Foundation. Abdominal CT scans were performed in dorsal or ventral recumbency with a voltage of 120 kV, slice thickness of 2 mm, pixel sizes ranging from 0.15 × 0.15 mm to 0.84 × 0.84 mm (Additional file 1) and a pitch of 1171. An automatic current selection function (DoseRight-D-DOM, Philips Medical Systems DMC GmbH, Hamburg, Germany) modulated current during tube rotation, which resulted in different mAs-products due to the patient’s body symmetry change. Patients were anaesthetised with levomethadon (L-Polamivet 0.2 mg/kg; CP-Pharma Handelsgesellschaft mbH, Burgdorf, Germany), diazepam (Ziapam®, 0.5 mg/kg, Laboratoire TVM, Lempdes, France) and propofol (individual dose depending on dose response; Narcofol® CP-Pharma Handelsgesellschaft mbH, Burgdorf, Germany) in accordance with the anaesthesia chart of the Small Animal Clinic of the University of Veterinary Medicine Hannover, Foundation. Inhalation anaesthesia with isoflurane (Isofluran CP®, CP-Pharma Handelsgesellschaft mbH, Burgdorf, Germany) was used during CT examination to maintain anaesthesia. A power injector (MedRad Vistron CT® 610 System, MedRad Inc., Indianola, USA) administered a non-ionic iodinated contrast agent (Xenetix® 300, Guerbet GmbH; Sulzbach, Germany, 2 mL/kg; flow rate: max. 3 mL/sec; duration: max. 30 s) into the vena cephalica antebrachii or vena saphena lateralis. No negative side effects were observed during anaesthesia and CT examination.

CT datasets were filed in DICOM format and analysed with an image-processing workstation (Extended Brilliance Workspace, Philips Medical Systems Inc., Ohio, USA). Prostatic volumes were evaluated with specific software (Amira 6.2; FEI, part of Thermo Fisher Scientific Inc., Hillsboro, Oregon, USA).

### Formula-based measurement

**Fig. 7:**
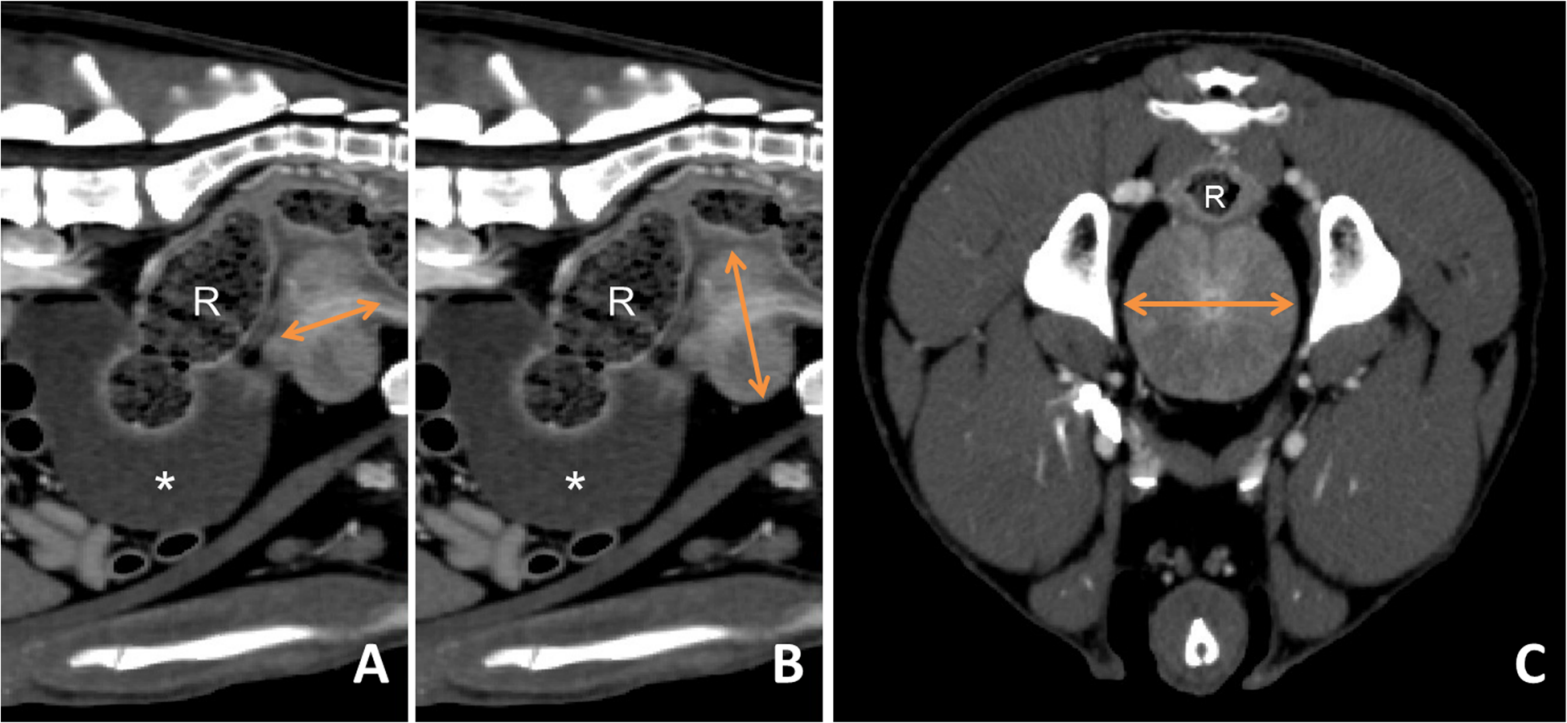
Formula Method: Measurements of **a** length in sagittal view, **b** height in sagittal view and **c** width in transversal view. Arrows = measurements of length, height and width, R = rectum, * = urinary bladder

Prostatic volume was determined using a formula for ellipsoid bodies (L x H x W/ $\frac{1}{6}$ π). Length, height and width of the prostate gland were measured in millimetres. In sagittal view, a median slice of the prostate was adjusted where the urethra was seen to cross the prostate gland. On this sagittal slice, the length of the prostate was defined as the maximum dimension from entry to exit of the urethra by means of a measuring line (Fig. 7a). Prostate height was measured in the same sagittal view, taking the largest extension of the organ in dorso-ventral dimension perpendicular to the prostatic length into account. For measuring the width, transversal slices were searched for the greatest latero-lateral extension of the prostate. A measuring line was drawn on the largest latero-lateral dimension crossing the intraprostatic urethra. Measurements and volumetric rendering were performed three times and the average (+/− standard deviation) of the three volume values was calculated. Hereafter, this method is termed “Formula” (Fig. 7).

### Wrap-based measurement

**Fig. 8:**
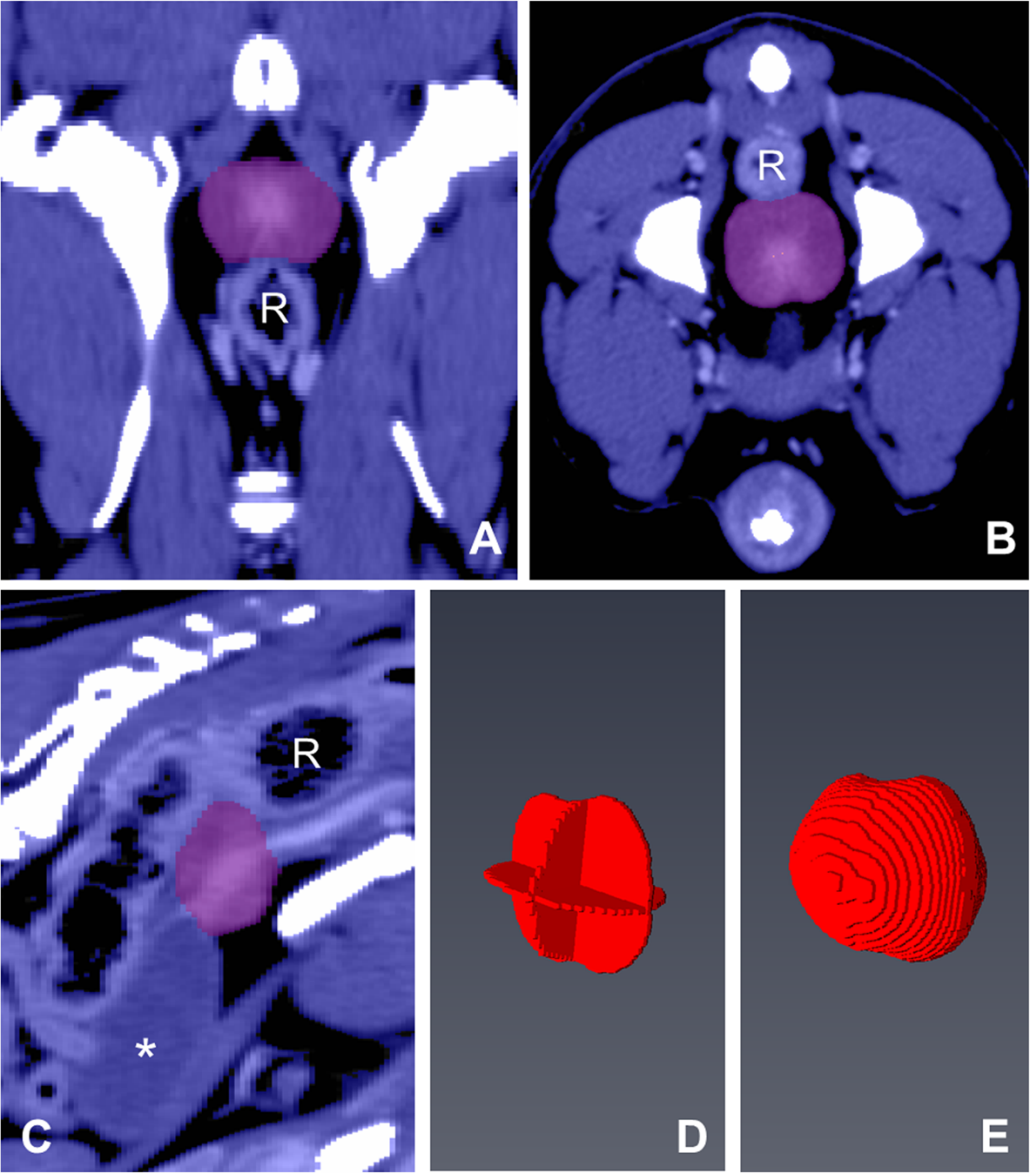
Wrap Function: The most central slice of the prostate gland is marked (purple) in **a** dorsal, **b** transversal and **c** sagittal view. Image **d** shows the three-dimensional cross-hair and image **e** represents the 3D reconstruction of the prostate gland using the Wrap function. R = rectum, * = urinary bladder

The “Wrap”-function of Amira was used to calculate the prostatic volume nearly automatically. For this purpose, transversal, sagittal and dorsal image stacks including the prostate gland were searched for the most central slice of the organ for each direction. In these planes, the prostate gland was marked manually with a mouse cursor (Fig. 8a-c) resulting in a three-dimensional cross-hair (Fig. 8d). Geared to this three-dimensional cross-hair, the wrap function uses algorithms to reconstruct the prostate gland and further compute the prostate gland’s volume (Fig. 8e). Cysts sticking out of the prostate’s surface were included in the cross-hair as far as possible. Hereafter, this method is termed “Wrap”.

### SAT (slice addition technique)

In a previous study [5], the SAT was used to measure prostatic volume. In order to determine the prostatic volume by means of SAT using Amira, a segmentation of the gland from the surrounding tissue was necessary. For this purpose, the prostate gland was encircled manually with a mouse cursor in transversal image view within all slices. The urethra was not excluded from the measurements. By multiplying the number of segmented voxels with the size of a single voxel, the total prostatic volume was computed. These results were obtained and compared to measurements in the present study.

Since the accuracy of this method had been validated in a previous study [5], the measured volume was considered as real prostatic volume in the present study.

### Phantom and cadaver measurements

The accuracy of the Formula and Wrap was checked against measurements of differently shaped phantoms and cadaver prostates with known volumes or volumes determined by water displacement in accordance with the recently described method [5]. For the phantom measurements, three differently shaped balloons filled with water and contrast medium (known volume) as well as three different phantoms of nearly prostatic shape, made from modelling clay (real volume determined by water displacement as recently described) were scanned. Datasets were analysed with the Formula method and Wrap function using the Amira software (Additional file 2). Furthermore, five canine cadavers were scanned and the prostatic volume was evaluated by Amira as explained above (Additional file 2). The dog owners’ consent had been previously obtained. Afterwards, necropsy was carried out, the prostate gland was removed and the real volume was determined by water displacement.

### Statistics

Statistical analysis was carried out with SAS® Enterprise Guide® 7.1 (Statistical Analysis Software, Heidelberg, Germany). Normal distribution was analysed with the Kolmogorov- Smirnov test or Shapiro-Wilk test. Differences between the Formula and Wrap volumes to those measured by SAT were analysed with a Wilcoxon signed-rank test. *P*-values less than 0.05 were assumed to be statistically significant. The evaluation of accuracy of both Function and Wrap was performed by linear regression analysis and Bland-Altman-Plots with GraphPad Prism (Graphpad Software, Version 7, San Diego, CA, USA 2003).

## Supplementary information

**Additional file 1.** The table shows the patient data: sex (m = male, n=neutred), age and weight, as well as the the group affiliation for prostatic structure and volume. Results of prostatic volume measurements in 3 different methods are added.

**Additional file 2.** The table shows the actual volume of phantoms and the prostate glands exentered from cadavers. Furthermore their volume measured with three different methods is shown. In the prostate glands, sex (m=male, n=neutred) and weight of the deceased animal is added.

## Data Availability

All data generated or analysed during this study are included in this published article and its supplementary information files.

## Abbreviations

%: Per cent
3D: Three dimensional
C: Cystic
cm: Centimeter
cm^3^: Cubic centimeter
CPSE: Canine prostate specific esterase
CT: Computed tomography
DICOM: Digital Imaging and Communications in Medicine
e.g.: Exempli gratia
Fig.: Figure
g: Gram
H: Homogeneous
I: Inhomogeneous
i: Intact
kg: Kilogram
kV: Kilovolt
L: Length
mAs: Milliampere seconds
mg: Milligram
mL: Milliliter
mm: Millimeter
n: Neutered
ng: Nanogram
no.: Number
PSA: Prostate-specific antigen
SAT: Slice addition technique
SD: Standard deviation
sec.: Seconds
TRUS: Transrectal ultrasound
vg: Volume group
W: Width
